# RecFOR Is Not Required for Pneumococcal Transformation but Together with XerS for Resolution of Chromosome Dimers Frequently Formed in the Process

**DOI:** 10.1371/journal.pgen.1004934

**Published:** 2015-01-08

**Authors:** Calum Johnston, Isabelle Mortier-Barrière, Chantal Granadel, Patrice Polard, Bernard Martin, Jean-Pierre Claverys

**Affiliations:** 1 Centre National de la Recherche Scientifique, Laboratoire de Microbiologie et Génétique Moléculaires, Toulouse, France; 2 Université de Toulouse, Université Paul Sabatier, Laboratoire de Microbiologie et Génétique Moléculaires, Toulouse, France; Université Paris Descartes, INSERM U1001, France

## Abstract

Homologous recombination (HR) is required for both genome maintenance and generation of diversity in eukaryotes and prokaryotes. This process initiates from single-stranded (ss) DNA and is driven by a universal recombinase, which promotes strand exchange between homologous sequences. The bacterial recombinase, RecA, is loaded onto ssDNA by recombinase loaders, RecBCD and RecFOR for genome maintenance. DprA was recently proposed as a third loader dedicated to genetic transformation. Here we assessed the role of RecFOR in transformation of the human pathogen *Streptococcus pneumoniae*. We firstly established that RecFOR proteins are not required for plasmid transformation, strongly suggesting that DprA ensures annealing of plasmid single-strands internalized in the process. We then observed no reduction in chromosomal transformation using a PCR fragment as donor, contrasting with the 10,000-fold drop in *dprA* ^-^ cells and demonstrating that RecFOR play no role in transformation. However, a ∼1.45-fold drop in transformation was observed with total chromosomal DNA in *recFOR* mutants. To account for this limited deficit, we hypothesized that transformation with chromosomal DNA stimulated unexpectedly high frequency (>30% of cells) formation of chromosome dimers as an intermediate in the generation of tandem duplications, and that RecFOR were crucial for dimer resolution. We validated this hypothesis, showing that the site-specific recombinase XerS was also crucial for dimer resolution. An even higher frequency of dimer formation (>80% of cells) was promoted by interspecies transformation with *Streptococcus mitis* chromosomal DNA, which contains numerous inversions compared to pneumococcal chromosome, each potentially promoting dimerization. In the absence of RecFOR and XerS, dimers persist, as confirmed by DAPI staining, and can limit the efficiency of transformation, since resulting in loss of transformant chromosome. These findings strengthen the view that different HR machineries exist for genome maintenance and transformation in pneumococci. These observations presumably apply to most naturally transformable species.

## Author Summary

Homologous recombination (HR) is a widespread process which maintains genome integrity and promotes diversity. In bacteria, HR mends damaged DNA to ensure genome integrity and is also involved in transformation, a mechanism of horizontal gene transfer allowing acquisition of new genetic traits. HR is driven by recombinases, which are loaded onto single-stranded DNA by the recombinase loaders RecBCD and RecFOR for genome maintenance. DprA was recently proposed as another loader dedicated to transformation. During transformation, foreign DNA is taken up as single strands and integrated into the chromosome by HR. In this study, we show that RecFOR is not involved in transformation in *Streptococcus pneumoniae*. These results provide further support to the existence of different HR machineries dedicated to genetic transformation and genome maintenance in this pathogen. In addition, we show that transformation with chromosomal DNA generates chromosome dimers with unexpectedly high frequency, and that their resolution requires RecFOR and the site-specific recombinase XerS. In cells lacking these proteins, dimers persist and have a detrimental effect on the efficiency of transformation. Since the HR mechanisms leading to dimer formation are most likely conserved, this effect is presumably general to naturally transformable species.

## Introduction

Homologous recombination (HR) is crucial for both maintenance of genome integrity and generation of diversity across all kingdoms of life. HR initiates universally from single-stranded (ss) DNA and involves strand exchange between homologous DNA sequences, catalyzed by homologous recombinases. In bacteria, HR is involved in repair of damaged DNA to ensure genome integrity (reviewed in [1]), and is also a crucial step in genetic transformation, a widespread mechanism of horizontal gene transfer which allows acquisition of new genetic traits (reviewed in [2]). Transformation involves internalization of ssDNA fragments generated from exogenous double-stranded (ds) DNA substrate, which can be incorporated into the chromosome via HR. This process generally occurs during a short time window called competence, during which all the proteins required for internalization and integration of ssDNA are produced (reviewed in [2], [3]).

Search for homology between DNA sequences during both genome maintenance and transformation is catalyzed by the homologous recombinase RecA. This enzyme requires dedicated loaders to promote its loading onto target ssDNA (reviewed in [4]). The end goal of bacterial recombinase loaders is to produce a filament of ssDNA coated in RecA, allowing HR to occur. Two main loaders are involved in genome maintenance, RecBCD targeting RecA to double strand DNA breaks (DSB) and RecFOR targeting it to ssDNA gaps. The latter was suggested as important for the restart of stalled replication forks, where such gaps are produced [5]. A third loader, DprA, has recently been characterized through studies of the protein from *Streptococcus pneumoniae* as dedicated to genetic transformation [6], [7].

RecBCD is an enzyme complex best characterized in *Escherichia coli* (reviewed in [8], although studied homologues are present in *Bacillus subtilis* (AddAB, [9]) and *S. pneumoniae* (RexAB, [10]) amongst others. The RecBCD loader substrate is dsDNA and as transforming DNA is internalized as ssDNA, a RecBCD-like complex was not expected to be involved in chromosomal transformation [11], which turned out to be true for *S. pneumoniae* where RexAB proteins are dispensable for transformation [10].

Mutants lacking the RecFOR proteins have shown increased sensitivity to DNA-damaging agents in various bacterial species including *E. coli* [12], *B. subtilis* [13] and *Deinococcus radiodurans* [14]. Unlike RecBCD, there is no evidence that the RecFOR proteins form a tricomponent enzyme complex, although RecR does interact directly with both RecO and RecF [15], [16]. However, all three proteins were shown to be able to access ssDNA coated in the single-stranded DNA binding protein (SSB) from ssDNA gaps in the chromosome and facilitating the loading of RecA to this ssDNA [17]. Indeed, the ability to displace SSBs from target ssDNA is a key activity of recombinase loaders that target ssDNA.

The third loader, DprA, appears to be conserved in all transformable species [2]. In *S. pneumoniae*, as a loader dedicated to the transformation process, DprA can presumably displace SsbB, a competence-induced SSB [18], [19], and load RecA onto the transforming ssDNA, prompting RecA filamentation, search for homology [6] within the recipient chromosome and subsequent integration. Inactivation of *dprA* almost completely abolished transformation efficiency in pneumococci [20], which is likely due partly to its role as a transformation-dedicated RecA loader [6] and partly due to its role in protecting internalized ssDNA, which is rapidly degraded in its absence [20]. This reduction was observed for both chromosomal and plasmid transformation. DprA was recently shown to interact directly with RecA, a property which is presumably necessary for loading of RecA onto transforming ssDNA and as such was found to be crucial for transformation [7]. A similar role for DprA as a transformation-dedicated RecA loader was recently established in *B. subtilis* [21] although cells lacking DprA were less severely impacted, with a 2-log deficit of transformation observed [22], [23].

Despite the presence of DprA, studies in *B. subtilis* showed that inactivation of *recO* reduced efficiency of chromosomal transformation 2-fold [21], suggesting that RecFOR might be involved in loading RecA onto ssDNA during both genome maintenance and transformation. For the transformation of replicative plasmids, no loss of efficiency was observed in *B. subtilis recF* or *recR* mutants, while *recO* mutants were 25-fold less efficient [24]. It was suggested that RecO was crucial for reconstruction of an intact plasmid molecule, allowing strand annealing from two internalized ssDNA fragments [25]. Interestingly, DprA was also important for plasmid transformation in *B. subtilis*, with a 50-fold reduction in efficiency observed in a *dprA^-^* mutant [25]. Crucially, replicative plasmid transformation is RecA-independent in *B. subtilis* [24] but not in *S. pneumoniae* [26], possibly due to the degradation of internalized ssDNA in the absence of pneumococcal RecA.

Here, we assess the importance of RecFOR for transformation in *S. pneumoniae*. We show that pneumococcal RecFOR proteins play no role in chromosomal or plasmid transformation but are required for resolution of chromosome dimers occurring as intermediates in the formation of merodiploids by transformation [27]. We report that chromosome dimers are generated at an unexpected high frequency by self-transformation, as well as interspecies transformation, and that their proper resolution also requires XerS [28], a tyrosine recombinase related the XerCD tyrosine recombinase of *Escherichia coli* which catalyzes chromosome dimer resolution by site-specific recombination at *dif* sites [29]. We provide evidence that in the absence of RecFOR and/or XerS, dimers persist within a transformed population and can limit the efficiency of transformation, since resulting in the loss of transformant chromosomes.

## Results

### Impact of *recFOR* inactivation on pneumococcal cells

Each *recFOR* gene was inactivated by *mariner* mutagenesis, as previously described [30] and the effect of mutating these genes on pneumococcal cells was investigated (S1–S2 Text; S1–S2 Fig.; S1 Table). All mutants were found to have doubling times of between 42 and 47 min, slower than the wild type which doubled every 34 min (S1D Fig.). RecFOR proteins appeared involved in genome maintenance in *S. pneumoniae* as deduced from the extreme sensitivity of *recFOR* mutants to the alkylating agent methyl methanesulfonate and the DNA crosslinking agent mitomycin C (S2 Fig.).

### RecFOR and genetic transformation

To establish whether RecFOR played any role in transformation in pneumococci, we tested the ability of *recFOR* mutants to respond to signals inducing competence for genetic transformation. We analyzed the expression of a gene specifically induced during competence, *ssbB*, after addition of the competence-stimulating peptide (CSP) (S2 Text). Results show that single and double mutant cells respond to CSP with the same kinetics as wildtype cells (S1E Fig.).

We first determined that RecFOR are not involved in replicative plasmid transformation (S1 Text; S3 Fig.; S3 Table). We previously suggested that antagonization of plasmid transformation by SsbB could be due to direct inhibition of plasmid strand annealing by RecO [19]. However, inactivation of *ssbB* had the same effect in wt and *recO* ^-^ cells whether the concentration of donor DNA was high or low (S3A–B Fig.). These results confirm that RecO is not involved in annealing of internalized plasmid strands, a crucial step in reconstitution of intact plasmid molecules.

**Figure 1:**
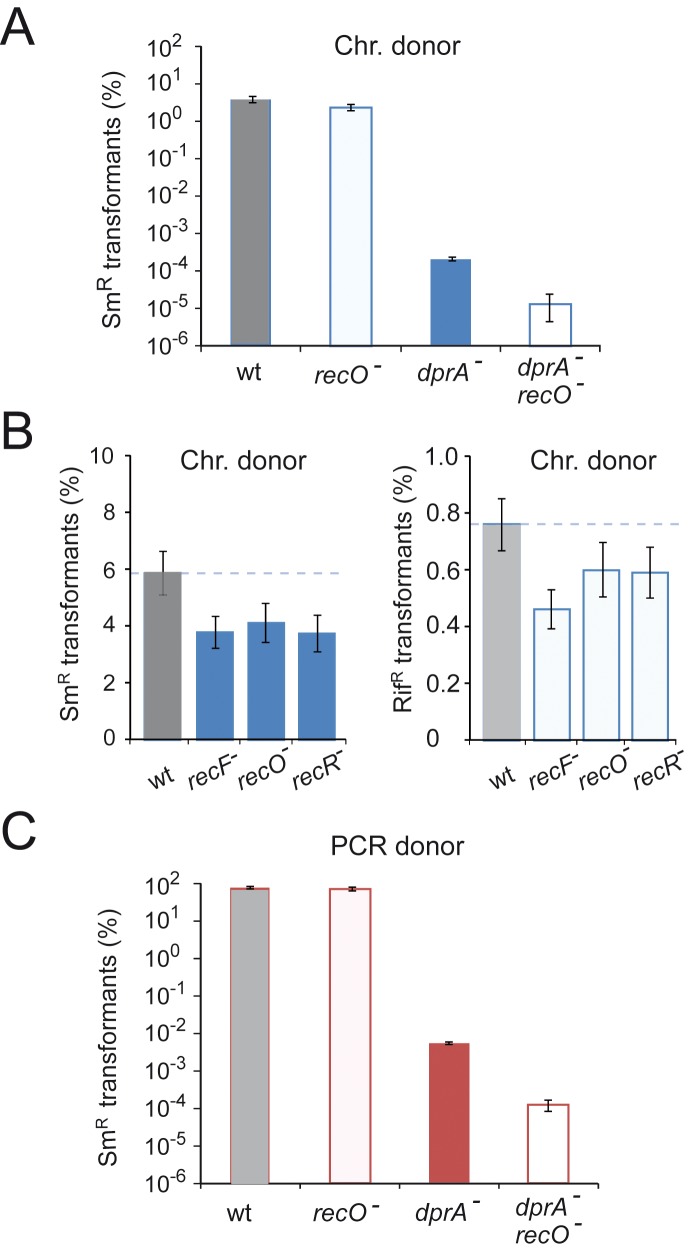
Impact of *recFOR* inactivation on transformation. (A) Chromosomal transformation frequencies in wildtype, *dprA*, *recO* and *dprA recO* mutant cells. Saturating concentration of R304 chromosomal DNA as donor (1 µg mL^−1^). Recipient strains: wild type, R1521; *recO* mutant, R3424; *dprA* mutant, R3426; *dprA*-*recO* double mutant, R3428. Error bars calculated from duplicate repeats. (B) Chromosomal transformation frequencies in wild type, *recF*, *recO* and *recR* mutant cells. Recipient strains: wild type, R1502; *recF* mutant, R2371; *recO* mutant, R2372, *recR* mutant, R2373. Donor DNA and selection for Str^R^ transformants as in panel A, as well as selection for Rif^R^ transformants. (C) Impact of *recO* inactivation on transformation with a unique PCR fragment as donor. Transformation with a 4.2-kb *rpsL41* (Str^R^) PCR fragment as donor (0.3 µg mL^−1^). Same recipient strains as in panel A. Error bars calculated from duplicate repeats.

To establish whether loss of *recO* impacted chromosomal transformation in pneumococci, we first compared transformation frequency of *recO* and *dprA* mutant cells. Transformation experiments with chromosomal DNA carrying the *rpsL41* point mutation conferring streptomycin resistance (Sm^R^) confirmed a major impact of *dprA* inactivation on chromosomal transformation, resulting in a ∼10,000-fold drop, while *recO* inactivation resulted in a limited deficit (∼1.6-fold drop; Fig. 1A). A further ∼40-fold drop was observed in two independent *recO dprA* double mutants (Fig. 1A), suggesting that the RecO protein can contribute to the processing of internalized ssDNA but mainly when DprA is absent. To confirm that *recFOR* inactivation had only a minor impact on chromosomal transformation, point mutations carried on chromosomal DNA were transformed into recipient cells either wildtype or lacking *recF*, *recO* or *recR*. Selection for integration of *rpsL41* and *rif23*, a point mutation conferring rifampicin resistance (Rif^R^), showed that both point mutations were accepted by *recFOR* mutants with efficiencies ∼70% of wildtype (1.45-fold average drop; Fig. 1B). Though reproducible, this difference appeared limited, suggesting that the RecFOR proteins play no major role in pneumococcal chromosomal transformation. This point is further examined and clarified in next sections of Results. It is of note that the results obtained with *rif23*, a mutation known to be targeted by the Hex mismatch repair system during pneumococcal transformaton [31], [32] allowed us to conclude that loss of RecFOR proteins does not affect mismatch repair (S1 Text; S2 Table).

The presence of a few residual transformants in the *recO dprA* double mutant (Fig. 1A) suggested that in the absence of both DprA and RecFOR loaders, RecA is sometimes capable of self-loading on internalized ssDNA. To confirm this conclusion, the transformation experiment was repeated but using as donor a PCR fragment carrying the *rpsL41* mutation to increase transformation efficiencies as a result of the use of a homogenous DNA preparation in which every fragment carries the genetic information under selection (Fig. 1C). The results paralleled those obtained with total chromosomal DNA, confirming a ∼40-fold drop in two independent *recO dprA* double mutants compared to the *dprA* single mutant. However, the >10-fold increase in transformants compared to chromosomal DNA in the double mutant relieved any ambiguity, confirming the appearance of transformants in the absence of both RecA loaders.

Surprisingly, the comparison of transformation frequencies for the *rpsL41* mutation revealed no difference between wildtype and *recO* ^-^ cells with a PCR fragment as donor (Fig. 1C), in contrast to the results when the donor was chromosomal DNA (Fig. 1A–B), leading us to conclude that RecO is not involved in the process of chromosomal transformation itself in *S. pneumoniae*.

### RecO and the generation of merodiploids by transformation

**Figure 2:**
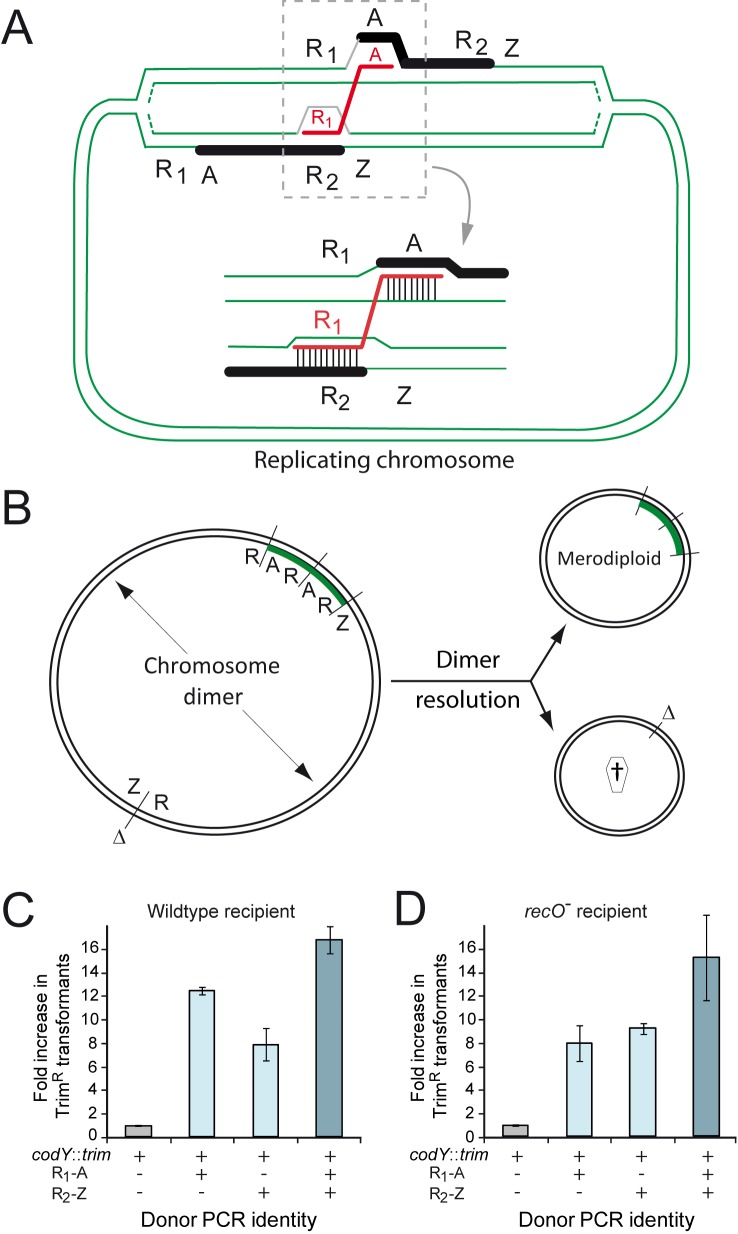
RecO and the generation of merodiploids by transformation. (A) Diagrammatic representation of the formation of merodiploids by transformation. This model involves ‘alternative pairing’ of a repeat sequence (R_1_) within the transforming ssDNA, i.e. pairing not with its chromosomal counterpart but with a similar repeat (R_2_) on one arm of a partially replicated recipient chromosome, coupled with ‘normal pairing’ of the non-repeat flanking ssDNA (A) on the other chromosome arm (next to the true chromosomal counterpart of R_1_). This bridges the two chromosome arms, creating a chromosome dimer. It is of note that this dimer differs from 'simple' chromosome dimers made of two directly repeated monomers. Resolution of this 'rearranged' chromosome dimer generates one merodiploid chromosome with the region between repeats duplicated and another chromosome lacking this region [27] (panel B). (B) Chromosome dimer resolution can be mediated by XerS or by homologous recombination, where RecA could be loaded by RecO. The duplicated region is shown in green. Δ, deletion; †, abortive chromosome. (C) Stimulation of merodiploid formation by transformation in wildtype cells (R246). (D) Stimulation of merodiploid formation by transformation in *recO* mutant cells (R3170).

While our results indicated that RecO was required neither for chromosomal nor for plasmid transformation, we remained intrigued by the ∼1.5-fold drop in transformation frequency observed in a *recO* mutant transformed with chromosomal DNA (Fig. 1A–B). We recently established that transformation stimulates the formation of partial chromosomal duplications, or merodiploids [27]. A single ∼3-kb DNA fragment partly repeated in the chromosome (see below) was sufficient to trigger formation of tandem-duplications ranging from ∼100 to ∼900 kb in size at various chromosomal locations. A mechanistic model for the formation of merodiploids was proposed and validated in that study [27]. Key to this model is the creation of a chromosome dimer as an intermediate, resolution of which generates one merodiploid chromosome and another chromosome lacking this region (Fig. 2A–B). We hypothesized that RecO was crucial for dimer resolution and that persistence of dimers in the transformed population could lead to chromosome or cell death, thus accounting for the observed drop in transformation of *recO* ^-^ cells.

To check this hypothesis, we investigated the formation of merodiploids induced by transformation in parallel in wildtype and *recO* mutant cells. The experimental set up for measurement of transformation-induced merodiploids involves the use a donor DNA mixture containing a merodiploid trigger fragment and a merodiploid scoring cassette. The merodiploid trigger, R_1_A or R_2_Z, is a ∼3-kb DNA fragment in which R_1_ and R_2_ correspond to repeats in the chromosome, and A and Z their respective non-repeated flanks. Once R_1_A is internalized, ‘alternative pairing’ of R_1_, defined as pairing with R_2_ on one arm of a partially replicated recipient chromosome, coupled with ‘normal pairing’ of A on the other chromosome arm (i.e. pairing with its couterpart next to R_1_ in the recipient chromosome) bridges the two chromosome arms (Fig. 2A) creating a dimer and, after resolution, a merodiploid (Fig. 2B). A 107 kb region originally flanked by R_1_ and R_2_ repeats is duplicated (referred to as duplication #1 [27]) and roughly represents minutes 39–43 if the chromosome is represented as a clock face. It includes the *codY* gene, which has been shown to be essential in *S. pneumoniae* [33]. The merodiploid scoring cassette, conferring resistance to trimethoprim (Trim^R^), is inserted in *codY* and thus can give rise to viable transformants only if this locus is duplicated, therefore selecting directly for merodiploid clones. A parallel control transformation with only the merodiploid scoring cassette allows measurement of background merodiploids spontaneously formed in the recipient culture.

Using this experimental set up, similar factors of stimulation of merodiploid formation by transformation were observed for each donor DNA set (*codY*::*trim* plus R_1_-A, R_2_-Z, or a mixture of R_1_-A and R_2_-Z) in wildtype and *recO* mutant cells (Fig. 2C–D). These results were consistent with RecO playing no role in the steps leading to formation of a chromosome dimer by transformation, which was not surprising since these steps are essentially identical to those involved in ‘classical’ chromosomal transformation, for which RecO is not required (Fig. 1C).

### Genetic evidence that RecO is necessary for resolution of chromosome dimers

**Figure 3:**
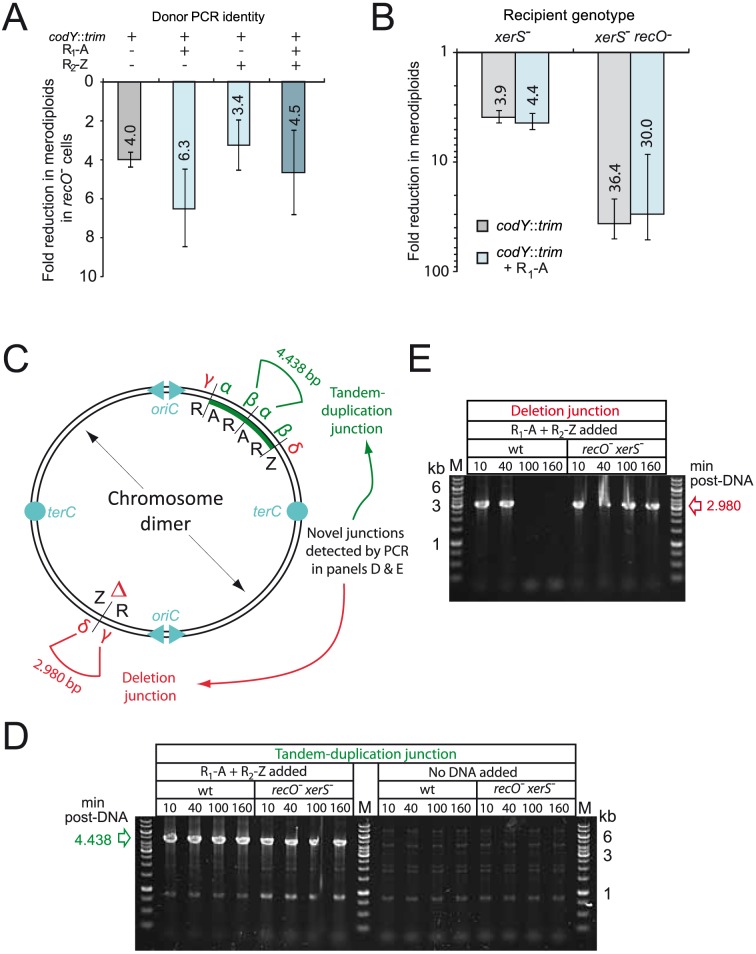
RecO and XerS are involved in pneumococcal chromosome dimer resolution. (A) Fold reduction in merodiploids formed in *recO* mutant compared to wildtype cells. Merodiploid formation was triggered using the R_1_-A PCR fragment as donor and the *codY*::*trim* donor cassette was used to score merodiploid transformants. Recipient strains used: wildtype, R246; *recO^-^*, R3170. (B) Fold reduction in merodiploids formed in *xerS* and *recO xerS* mutant compared to wildtype cells. Same experimental set up as in panel A. Recipient strains used: *xerS^-^*, R3214; *xerS^-^ recO^-^*; R3873. Since merodiploid frequency was low in the double mutant, representing few clones, we confirmed on a few randomly chosen clones that they were indeed merodiploid by detection of the tandem-duplication junction by PCR (panel D), as well as confirming that they had retained the antibiotic sensitivities associated with the parent. (C) Schematic of chromosome dimer and novel tandem duplication and deletion junctions detected by PCR. α and β represent primers used to detect the novel junction created by tandem duplication. δ and γ represent primers used to detect the deletion junction created as a consequence of tandem duplication. Both junctions are present on the chromosome dimer, while the merodiploid chromosome possesses only the tandem-duplication junction, and the abortive chromosome possesses only the deletion junction. (D) Detection of tandem-duplication junction on cultures of wildtype or *recO^-^ xerS^-^* cells transformed with either R_1_-A and R_2_-Z PCR fragments or no DNA. PCRs carried out at different time-points after DNA addition with primers CJ242 and merod-b to amplify a 4,438 bp DNA fragment diagnostic of the tandem duplication. (E) Detection of deletion junction on same cells as in panel A (DNA added only). PCRs carried out with primers CJ244 and CJ245 to amplify a 2,980 bp DNA fragment diagnositic of the deletion junction. Strains used in both panels: wildtype; R246, *recO^-^ xerS^-^*; R3873.

While inactivation of *recO* did not affect the stimulation factor, the absolute frequency of merodiploids, either spontaneously present or triggered by partly repeated donor fragment(s) was reduced by 3.4 to 6.3-fold in *recO* ^-^ cells (Fig. 3A). This finding provided support to the hypothesis that RecO is involved at a later stage in the formation of merodiploids, namely the resolution of chromosome dimers required to generate a merodiploid chromosome (Fig. 2B). Two mechanisms are envisioned for resolution, one involving HR via RecA and potentially requiring the assistance of RecO for loading, and the other mediated by the site-specific recombinase XerS acting at *dif* sites [28]. To further document the resolution of chromosome dimers in *S. pneumoniae*, we repeated the experiment in *xerS^-^* and *recO^-^ xerS^-^* cells. The *xerS* mutant displayed a general loss of merodiploid formation of ∼4-fold (Fig. 3B), similar to a *recO* mutant. Interestingly, the *recO xerS* double mutant showed an even greater loss, of around 35-fold (Fig. 3B), suggesting that >95% of chromosome dimers could not be resolved in the absence of both RecO and XerS proteins. The similar effect of *xerS* and *recO* inactivation, and the cumulative effect of *recO* and *xerS* mutations on merodiploid formation provided strong support to our hypothesis that RecO is involved in chromosome dimer resolution and indicated that RecO and XerS are both required for proper resolution.

### Physical evidence that both RecO and XerS are required for chromosome dimer resolution

To obtain physical evidence in support of our conclusions, we firstly confirmed that a wildtype level of dimeric chromosomes was indeed produced in the absence of RecO (and XerS), by monitoring the presence of the novel junctions predicted in the chromosome dimer by PCR (i.e. both tandem-duplication and deletion junctions; Fig. 3C–E) on wildtype and *recO^-^ xerS^-^* populations transformed with R_1_-A PCR fragment. The tandem-duplication junction was detected similarly in transformed wildtype and double mutant cells, at different time points after addition of transforming DNA (10–160 min; Fig. 3D). Note that weak bands due to low level spontaneous merodiploid formation were detected in the no-DNA controls, at the same time points. This result indicated that chromosome dimers are formed with similar efficiency in wildtype and *recO^-^ xerS^-^* cells, suggesting no role for RecO in the formation of dimeric chromosomes both during normal growth and by transformation.

Secondly, we confirmed the disappearance of the deletion junction at 100 min in wildtype cells (Fig. 3E), which is consistent with our previous report [27] and readily explained by resolution of the chromosome dimer in the wildtype background and loss of cells with the abortive chromosome missing a large region of 107 kb, including the essential *codY* gene (Fig. 2B; referred to as deletion #1 hereafter). In contrast, persistence of deletion #1 junction in *recO^-^ xerS^-^* cells (Fig. 3E) provided direct evidence that as suggested above on the basis of genetic data (Fig. 3B), these mutant cells did not properly resolve >90% of chromosome dimers. This supports our conclusion that RecO is involved at a late stage in merodiploid formation, rather than in transformation itself, which is dependent on DprA.

Further physical evidence for the persistence of deletion junctions and therefore of chromosome dimers in *recO^-^ xerS^-^* cells was obtained by investigating another two merodiploid chromosomes previously shown to form during transformation with total genomic DNA (31). These merodiploid chromosomes contained a duplication of 144 kb (site #2, 10–15 minutes on the chromosome) and 210 kb (site#3, 23–29 minutes on the chromosome) respectively. The deletion junctions (#2 and #3) of the reciprocal products generated through resolution of the parental dimeric chromosome were found to disappear with similar kinetics in wildtype cells, while persisting in *recO^-^ xerS^-^* cells (S5 Fig.).

### Growth phase impacts the kinetics of chromosome dimer resolution

We previously observed that the deletion junction #1 had disappeared at 100 min post DNA addition in wildtype cells, as confirmed in this study (Fig. 3E and S5 Fig.), but was still readily detectable after 70 min [27]. We were intrigued by the finding that even the basal level of deletion junctions produced by spontaneous merodiploid formation disappeared at 100 min (Fig. 3E and S5 Fig.), suggesting a general phenomenon leading to death of all cells harboring abortive chromosomes in the culture.

**Figure 4:**
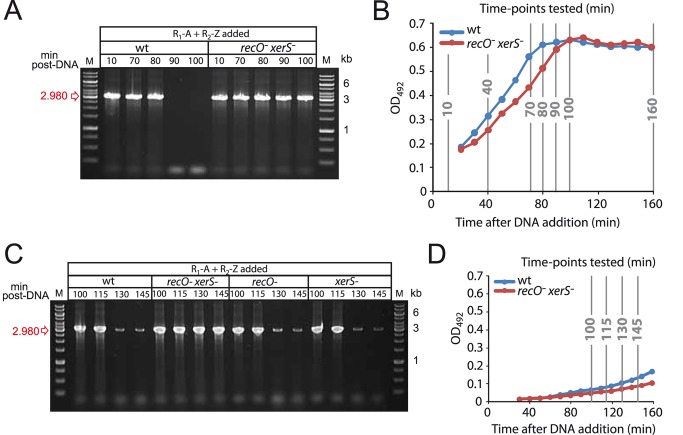
Growth phase impacts the kinetics of deletion-junction loss in transformed cultures. (A) Experiment in Fig. 3D repeated with different time-points after DNA addition. (B) Growth curves representing growth of wildtype and *recO^-^ xerS^-^* cells during experiment in Fig. 3D and panel A. (C) Repeat of experiment in panel A but with cells diluted 10-fold 20 min after DNA addition, and later time points taken. *xerS* and *recO* mutants were included to determine the kinetics of deletion junction disappearance in these two backgrounds. (D) Growth curves representing growth of wildtype and *recO^-^ xerS^-^* cells during experiment in panel C. Strains used in all four panels: wildtype; R246, *recO^-^ xerS^-^*; R3873.

To determine whether chromosome dimer resolution was impacted by growth phase, we repeated the experiment with deletion #1 junction, determining more precisely when deletion junctions could no longer be detected by focusing on time-points between 70 and 100 min post-DNA addition, while monitoring in parallel the growth of the culture. Results show that the both spontaneous and transformation-induced deletion junctions disappear between 80 and 90 min (Fig. 4A), which correlates with entry to stationary phase (Fig. 4B). Note that although *recO^-^ xerS^-^* cells display slightly delayed entry to stationary phase, this can by no means explain the failure to detect junction loss after 160 min in Fig. 3E.

To confirm that entry to stationary phase was somehow dictating deletion junction loss, we changed the experimental set up used in this and a previous [27] study so as to delay entry of transformed cells into the stationary phase. Cells were diluted 20 min after DNA addition, and the presence of the deletion junction was investigated at later time points in wildtype, *recO^-^*, *xerS^-^* and *recO^-^ xerS^-^* cells. Loss of transformation-induced deletion junction was not detected prior to 130 min, indicating that the growth phase had a net impact on the process (Fig. 4C–D). Note that basal level spontaneous duplications still occurred, explaining the weak bands observed at 130 and 145 min. Identical kinetics were observed for single *recO^-^* and *xerS^-^* mutants suggesting that dimers can be resolved in these mutants. However, the deletion junction persisted in *recO^-^ xerS^-^* cells, showing again that these are unable to resolve chromosome dimers.

### Frequent chromosome dimerization and defective resolution accounts for decreased transformation frequency in *recO* ^-^ as well as *xerS* ^-^ cells

These results were consistent with our working hypothesis that RecO is crucial for dimer resolution and that persistence of dimers in the transformed population leads to chromosome or cell death. However, to account for the observed ∼1.5-drop in transformation frequency of a point mutation in *recO* ^-^ cells, our hypothesis necessarily implied that formation of chromosome dimers during transformation with total genomic DNA occurred at unexpectedly high frequency. To result in such a drop, this type of event should occur in 30–35% of the cells, being efficiently resolved and therefore remaining ignored in wild type but not in *recO^-^* cells. In the latter, co-transformation of the fragment carrying the selected marker (e.g. the *rpsL41* mutation) and a merodiploid trigger fragment would result in the loss of the potential Sm^R^ transformant.

**Figure 5:**
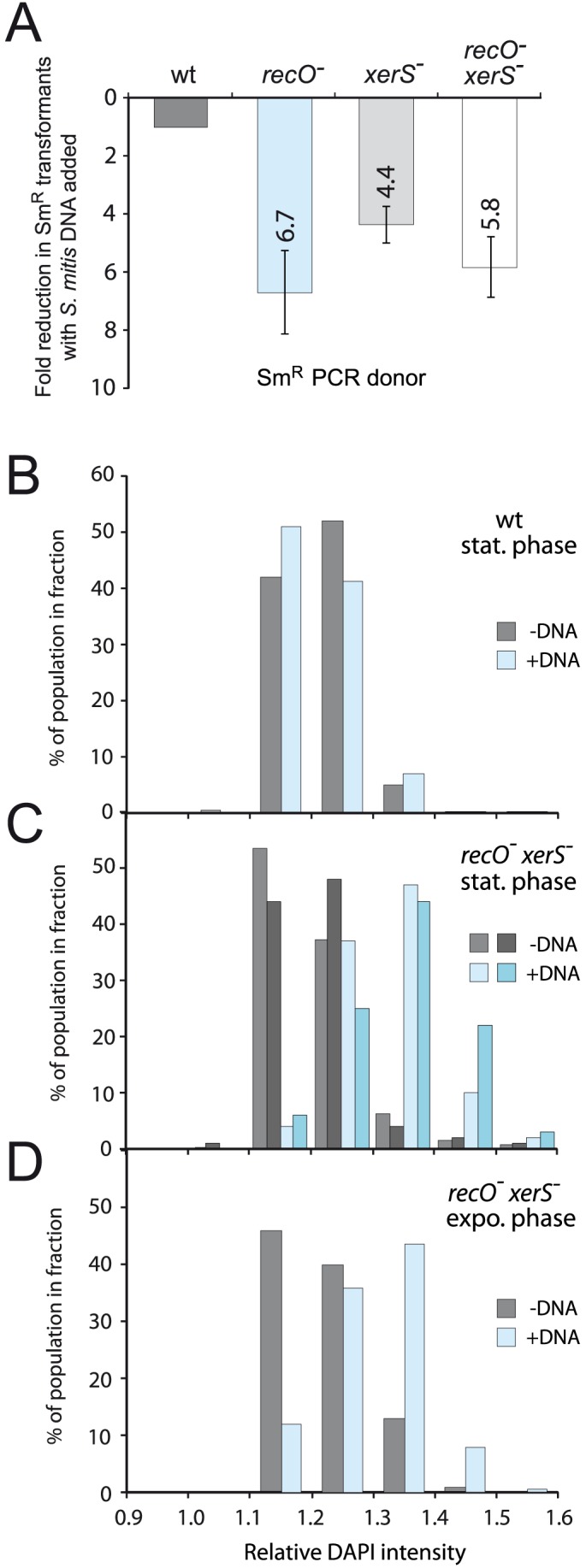
High frequency formation of chromosome dimers upon transformation with *S. mitis* chromosomal DNA. (A) *S. mitis* DNA impacts transformation frequency of *recO*, *xerS* and *recO xerS* mutant cells. Comparison of transformation efficiency using as donor a mixture of PCR fragment carrying the *rpsL41* mutation (Sm^R^) and *S. mitis* chromosomal DNA. As a slight competition for uptake is observed between the PCR fragment and *S. mitis* chromosomal DNA in wildtype cells (reducing efficiency compared to PCR fragment alone by 1.6-fold), we adjusted fold reduction in wildtype to 1 and normalized to this same factor in all mutant cells. PCR concentration: 1.5 ng mL^−1^; chromosomal DNA concentration: 1 µg mL^−1^. Strains used: wildtype; R246, *recO^-^*; R3170, *xerS^-^*; R3214, *recO^-^ xerS^-^*; R3873. (B) Monitoring ploidy through DAPI staining in cells transformed with *S. mitis* chromosomal DNA and grown to stationary phase (blue bars), compared to non-transformed competent cells (grey bars). Samples were taken at 160 min (see Fig. 4B). No difference in DAPI fluorescence intensity profile was observed in wildtype cells. Relative fluorescence intensity calculated by dividing total cell intensity by cell area. Results shown are representative of three individual experiments. Strain used: wildtype, R246. (C) Monitoring ploidy through DAPI staining in *recO^-^ xerS^-^* cells transformed with *S. mitis* chromosomal DNA and grown to stationary phase. Two individual sets of data showing with non-transformed cells (light and dark grey) and transformed cells (two shades of blue). A shift in intensity profile towards greater fluorescence was observed with cells transformed with *S. mitis* DNA (compare blue and grey bars). Sampling and analysis as in panel B. Strain used: *recO^-^ xerS^-^*, R3873. (D) Monitoring ploidy through DAPI staining in *recO^-^ xerS^-^* cells transformed with *S. mitis* chromosomal DNA and maintained in exponential growth (blue bars), compared to non-transformed competent cells (grey bars). Samples were taken at 80 min (see Fig. 4D). A shift in intensity profile towards greater fluorescence was observed with cells transformed with *S. mitis* DNA (compare blue and grey bars). Same calculations and strain as in panel C.

On the basis of this explanation, we predicted that any transforming DNA capable of inducing chromosome dimerization at higher rate than pneumococcal DNA should further decrease the loss of transformants in *recO* mutant cells. *Streptococcus mitis* B6 chromosomal DNA represented an ideal tool to check our prediction as this species is a close relative of *S. pneumoniae*, therefore allowing homologous exchanges, but harboring an overall genome arrangement with a striking X-alignment when compared to pneumococcal genomes, indicative of many symmetrical inversions [34]. Every fragment overlapping an inversion site is predicted to generate a chromosome dimer upon integration into the pneumococcal chromosome. We therefore used as donor in transformation of wildtype and *recO* ^-^ cells a mixture of the *rpsL41* PCR fragment and *S. mitis* B6 chromosomal DNA. Results show that while co-transformation with *S. mitis* DNA had no effect on the frequency of transformants for the *rpsL41* PCR fragment in wildtype cells, it reduced this frequency by 6.7-fold in *recO* mutant cells (Fig. 5A).

To establish that this reduction was only due to the failure to properly resolve the chromosome dimers formed by co-transforming *S. mitis* DNA, this experiment was reproduced with *xerS* mutant cells. The results revealed a 4.4-fold reduction (Fig. 5A) providing strong support to our interpretation since XerS as a site-specific recombinase is not expected to have any effect on chromosomal transformation, except if chromosome dimers are generated in the process, requiring its action. A similar reduction, 5.8-fold, was observed in a control experiment with *recO xerS* double mutant cells (Fig. 5A). Considering a 5.6-fold average reduction in Sm^R^ transformants, we conclude that chromosome dimers are formed in ∼80% of the cells upon co-transformation with *S. mitis* chromosomal DNA. It is of note that if chromosome dimers do not form in ∼1 cell out of 5, this would readily account for the fact that no greater decrease in Sm^R^ transformants is observed in the *recO xerS* double mutant compared to the single mutants. In other words, if 20% of Sm^R^ transformants are chromosome-dimer free, this would *de facto* limit the maximum drop in transformation frequency observable in the *recO xerS* double mutant to ∼5-fold, as dimer-free transformants would obviously survive.

While these results were consistent with a high incidence of chromosome dimers in the transformed population, none of the above experiments, including those monitoring the presence of the novel junctions predicted in the chromosome dimer by PCR, detected the presence of a dimer per se. In an attempt to obtain more direct evidence for the presence of chromosome dimers, we stained cells transformed with *S. mitis* DNA with DAPI (4',6-diamidino-2-phenylindole) to quantify their DNA content. We tested whether we could detect a difference in cell fluorescence between wild type and *recO^-^ xerS^-^* cells in stationary phase, when unresolved dimers should be present in the latter (Fig. 4A–B). While there was no difference in fluorescence in wild type cells whether they had been transformed with *S. mitis* DNA or not (Fig. 5B), a clear increase in intensity was observed in transformed *recO^-^ xerS^-^* cells (Fig. 5C–D), fully consistent with persistence of chromosome dimers within these cells. These results provide strong support to our hypothesis that a high frequency of chromosome dimers are formed by transformation, with RecFOR and XerS required for proper resolution.

## Discussion

### RecFOR and the maintenance of pneumococcal genome

As expected from studies in other species, the RecFOR proteins appear important for pneumococcal genome maintenance, as deduced from the impact of inactivation of the corresponding genes on growth rate (S1 Fig.) and sensitivity to both methyl methanesulfonate and mitomycin C (S2 Fig.). This extreme sensitivity is reminiscent of that of a pneumococcal *recA* mutant [35]. On the other hand, quite unexpectedly, the RecFOR proteins appear involved in another facet of pneumococcal genome maintenance revealed by investigation of the impact of *recO* inactivation on the formation of merodiploids triggered by transformation. This analysis established a requirement for RecO for proper resolution of chromosome dimers (see below), which to the best of our knowledge had so far not been documented in any bacterial species.

### RecO is not required for plasmid transformation

The pneumococcal RecFOR proteins appear not to be required for plasmid transfer by transformation (S1 Text). This contrasts with the situation in another model transformable species, *B. subtilis*, in which plasmid transformation was reduced by almost 2-logs in a *recO* mutant [21]. It was suggested that RecO was crucial for reconstruction of an intact plasmid molecule, allowing strand annealing from two internalized ssDNA fragments in this species [25]. In *S. pneumoniae*, we previously observed that the competence-induced single-stranded DNA-binding protein SsbB, which is dedicated to chromosomal transformation, antagonized plasmid transformation at a high concentration of donor plasmid DNA [19]. We tentatively attributed the inhibitory effect of SsbB to antagonization of RecO annealing of plasmid single strands [19]. However, plasmid transformation carried out in this study revealed that inactivation of *ssbB* resulted in a similar increase in transformation in both wildtype and *recO* mutant cells (S3A Fig.). We conclude that the increase in plasmid transformation in the absence of SsbB is not due to a relief of inhibition of RecO-dependent annealing of internalized plasmid single-strands and that RecO is not involved in plasmid strand annealing in pneumococcal transformation. We suggest DprA as an obvious candidate based on the stimulation of the annealing of complementary strands by purified DprA previously observed in vitro [6]. Though the observation that plasmid transformation is abolished in *dprA* mutant cells [20] would be consistent with this proposal, it is inconclusive because in *S. pneumoniae* internalized ssDNA is very rapidly degraded in cells lacking DprA [20]. On the other hand, the finding that plasmid transformation is reduced by 2-logs in a *dprA* mutant of *B. subtilis* [21], where DprA is not required for protection of internalized ssDNA, is fully consistent with a role of DprA in plasmid strand annealing. Furthermore, the observation of an only 2-fold reduction in plasmid transformation in a *recO*-*recA* double mutant [21], implying efficient plasmid strand annealing, makes DprA an obvious candidate to promote annealing, at least in *B. subtilis* cells lacking RecO and RecA.

### RecO is also not required for chromosomal transformation

As concerns chromosomal transformation, we could not detect any effect of the inactivation of *recFOR* on the efficiency of Hex-dependent mismatch repair of point mutations, occurring at the heteroduplex stage during integration of transforming DNA (S1 Text). Chromosomal integration of a heterologous cassette was also unaffected in a *recO* mutant (S4 Fig.). On the other hand, we repeatedly observed a limited (∼1.45-fold) but significant reduction in the frequency of transformation of a point mutation when genomic DNA was used as donor in transformation of *recFOR* mutant cells (Fig. 1B). However, the direct comparison of transformation frequencies of wildtype and *recO* ^-^ cells for a point mutation but using a PCR fragment as donor revealed no difference (Fig. 1C). The latter finding leads us to conclude that RecO is not involved in the processing of transforming DNA in wildtype pneumococci.

A number of studies have previously explored the role of RecO in chromosomal transformation in other naturally transformable species. Mirroring our results, RecO was shown to play no role in transformation in *Neisseria gonnorhoeae* [36] and *Helicobacter pylori* [37], [38]. A slight reduction in transformation efficiency was observed in *Deinococcus radiodurans recO* ^-^ cells, and authors concluded that RecFOR was the major loader of RecA onto ssDNA during transformation [39]. Nevertheless, as DprA is present in *D. radiodurans* and though its role in transformation has not been determined, we suggest that this protein, and not RecFOR, is the main loader of RecA during transformation in this species as well. In any case, the finding that RecO is also not required for chromosomal transformation in wildtype pneumococci and most other tested species is fully consistent with the view that the transformation-dedicated loader DprA ensures the loading of RecA onto internalized ssDNA. Altogether, these results provide further support to the notion that different machineries exist in *S. pneumoniae*, as well as presumably in many other transformable species, to promote genetic transformation on one hand and ensure genome maintenance on the other.

### RecO, XerS and the resolution of chromosome dimers

We were intrigued by the ∼1.5-fold drop compared to wildtype observed when a point mutation was transferred from chromosomal DNA but not if the same mutation was transferred on a short PCR fragment (Fig. 1). As we previously showed that transformation with pneumococcal chromosomal DNA generates a variety of merodiploids, proceeding through the creation of a chromosome dimer which is subsequently resolved [27], we hypothesized that *recO^-^* cells were unable to properly resolve dimers, resulting in a loss of transformants in cells integrating both the fragment carrying the point mutation and a DNA fragment triggering chromosome dimerization. This hypothesis is supported by the observation of a reduction in merodiploid formation in *recO* mutant cells compared to wild type (Fig. 3A), as well as of a similar reduction in *xerS* mutant cells (Fig. 3B). The latter represents the first *in vivo* evidence of a function for pneumococcal XerS, even if this function was assumed due to identification of XerS as a tyrosine recombinase with a function potentially similar to the XerCD proteins in *Escherichia coli* [28].

Genetic evidence that both RecO and XerS are required for proper dimer resolution was provided by the >95% reduction in merodiploids in double mutant cells (Fig. 3B). This finding was corroborated by the observation that two PCR fragments detecting the new junctions (i.e. tandem-duplication and its accompanying deletion counterpart; Fig. 3C) present in the chromosome dimer persist in cells lacking both XerS and RecO (Fig. 3DE, 4A, 4C and S5 Fig.). This persistence is indicative of poor dimer resolution and provides physical evidence for the maintenance of unresolved chromosome dimers in the double mutant.

It is unclear what the mechanistic significance of the genetic requirement for both *recO* and *xerS* for chromosome dimer resolution is. Owing to the atypical nature of the pneumococcal site-specific recombination machinery, comprising a single component instead of two as in the *E. coli* paradigm, RecO might directly assist XerS for recombination at *dif*, possibly to help stabilize the synapsis complex. Alternatively, RecO could for example be needed because the RecFOR machinery for HR contributes to proper chromosome segregation after XerS-catalyzed site-specific recombination has taken place at the *dif* site. It is of note that despite a>95% reduction in merodiploid formation in *recO^-^ xerS^-^* cells, a few bona fide merodiploids were obtained (Fig. 3B), indicating that chromosome dimers could still be resolved, though with poor efficiency, in the absence of both RecO and XerS. This result implies the existence of another resolution pathway, which could possibly rely on RecA-driven HR, involving the RexAB loader (the pneumococcal counterpart of RecBCD [10]) to load RecA.

Interestingly, resolution of chromosome dimers as deduced from the loss of deletion junctions appeared faster in cells entering stationary phase compared to those remaining in exponential phase (Fig. 4). This loss occurred with similar kinetics irrespective of the nature of the deletion for transformation-induced merodiploids (Fig. 4 and S5 Fig.). It also concerned the deletion junctions produced by spontaneous merodiploid formation which completely disappeared in stationary phase (e.g. 100 min time point in Fig. 3E, 4A and S5 Fig.), suggesting a general phenomenon leading to death of all cells harboring abortive chromosomes. This 35–40 min delay in resolution (115–130 min compared to 80–90 min) in exponentially growing cells, which roughly corresponds to one generation, may not indicate that resolution occurs earlier in stationary phase cells but simply that dimer resolution occurred at the same time in both populations of cells, and that cells with an abortive chromosome survive longer in exponential conditions. Alternatively, it is possible that entry to stationary phase forces resolution of chromosome dimers so that none remain in quiescent stationary phase cells. As a consequence, cells with a deleted chromosome would be generated and die rapidly, explaining the complete loss of deletion junction in wildtype cells entering stationary phase. Whatever the underlying mechanism, this phenomenon potentially ‘cleanses’ a pneumococcal population of cells from any rearrangements leading to major deletions. It can therefore be predicted that a pneumococcal population coming out of stationary phase (or, more generally, non-dividing cells in nature) thus contains a minority of non-healthy cells.

### Defective resolution of chromosome dimers accounts for decreased transformation frequency in *recO* ^-^ as well as *xerS* ^-^ cells

The documented defect in resolution of chromosome dimers in *recO* ^-^ cells could account for the ∼1.5-fold drop in transformation of a point mutation only if dimers formed in 30–35% of cells transformed with *S. pneumoniae* genomic DNA, leading to loss of the transformed chromosome. In support of this interpretation, we predicted that any transforming DNA inducing a higher rate of chromosome dimerization than pneumococcal DNA should further decrease the loss of transformants in *recO* ^-^ cells. This prediction was verified using *S. mitis* B6 chromosomal DNA which harbors many symmetrical inversions compared to the *S. pneumoniae* chromosome [34], with every fragment overlapping an inversion site potentially generating a chromosome dimer upon integration into the pneumococcal chromosome. Co-transformation of *S. mitis* chromosomal DNA with a PCR fragment containing a point mutation caused a loss of ∼80% of potential transformants in *recO^-^* cells (Fig. 5A). Similar reductions were observed in *xerS^-^* cells (Fig. 5A). Since the only suggested role of XerS is in site-specific recombination to resolve chromosome dimers, this provides strong support to our hypothesis that a high frequency of chromosome dimers are formed by transformation, with RecFOR and XerS required for proper resolution. Furthermore, the use of DAPI staining to quantify DNA content revealed a clear increase in intensity in *recO^-^ xerS^-^* cells transformed with *S. mitis* DNA (Fig. 5CD), fully consistent with a high incidence of chromosome dimers persisting within these cells. Overall, these results establish that an inability to resolve chromosome dimers efficiently, and not a specific role in the transformation process, accounts for the decreased transformation efficiency observed in *recO^-^* cells when chromosomal DNA is used as donor.

### Significance of our observations for *S. pneumoniae* and other transformable species

While we attribute the decrease in transformation frequency in *S. pneumoniae recO* ^-^ cells to the defective resolution of chromosome dimers frequently formed during the process, it would be interesting to clarify whether a similar explanation accounts for the ∼2-fold loss of transformants in a *B. subtilis recO* mutant [21]. Interestingly, in *B. subtilis*, both *recO* and *addAB* (*B. subtilis* RecBCD homologue) mutant cells showed similar reductions in chromosomal transformation efficiency, while a double mutant showed a 10-fold reduction [24], [40]. This can be readily explained by high dimer formation during transformation with chromosomal DNA, which are poorly resolved in the absence of both loaders involved in genome maintenance. Furthermore, since merodiploid formation relies on classic recombination mechanisms and may be stimulated by transformation in a wide range of transformable species, it is likely that dimer formation is a common occurrence in cells transformed with chromosomal DNA.

Our observation that chromosome dimers frequently form via transformation is of practical importance when analyzing genetic transformation in *S. pneumoniae* by cell imaging. It is crucial to realize that a significant fraction of the population is engaged in the process of resolving chromosome dimers, via RecFOR, RecA and XerS proteins, and therefore to set up experimental conditions allowing a clear distinction between images corresponding to chromosomal transformation *per se* and those resulting from resolution of chromosome dimers subsequent to transformation. This cautionary note may also apply to *B. subtilis* if chromosomal transformation frequently produces dimers in this species as well (which is not unlikely since repeated sequences are also present in the chromosome, e.g. ribosomal operons). It would be interesting to clarify this point, as it may lead to reinterpretation of previous analyses. For example, a study showed the formation of so called ‘threads’ of RecA in cells transformed with chromosomal DNA, concluding that these structures may mediate homology search and strand invasion for genetic transformation [41]. Since such structures were seen in a fraction of transformed cells (24%), it is possible that dimer resolution mediated by HR (catalyzed by RecFOR and RecA) was being observed. Future work will determine whether our conclusion that RecFOR proteins are involved in genome maintenance but play no role in either chromosomal or plasmid transformation in *S. pneumoniae* also applies to the RecFOR proteins of other transformable species.

## Materials and Methods

### Bacterial strains, plasmids, primers, media and transformation

All the strains and plasmids used in this work are listed, together with primers, in S1 Table. Standard procedures for transformation and growth media were used [42]. Antibiotic concentrations (µg mL^−1^) used for the selection of *S. pneumoniae* transformants were: chloramphenicol (Cm), 4.5; erythromycin (Ery), 0.05–0.2; kanamycin (Kan), 250; rifampicin (Rif), 2; spectinomycin (Spc), 100; streptomycin (Sm), 200; tetracycline (Tet), 1. For the monitoring of growth and *luc* expression, precultures were gently thawed and aliquots were inoculated (1 in 100) in luciferin-containing [43] C+Y medium and distributed (300 ml per well) into a 96-well white microplate with clear bottom. Relative luminescence unit (RLU) and OD values were recorded throughout incubation at 37°C in a LucyI luminometer (Anthos).

### Detection of duplication and deletion junctions on transformed populations

To detect novel junctions produced by merodiploidy during transformation, experiments were carried out as previously described [27], with modifications. Briefly, recipient cells transformed with either pneumococcal chromosomal DNA or PCR amplifications of R_1_-A and R_2_-Z fragments, and 200 µL samples taken at varying time-points after DNA addition. Samples were centrifuged, and pellets resuspended in 10 µl C+Y medium with 15% glycerol. PCRs to detect various duplication and deletion junctions were done directly on 2 µL of these culture samples. Primers used for these PCRs are detailed in the appropriate Figure Legends.

### Quantification of DNA within cells by DAPI staining

To quantify the DNA content of cells, DAPI staining was used. Wild-type and *recO^-^ xerS^-^* cellswere grown to OD 0.1 in C+Y medium, and 25 ng mL^−1^ added. After 10 min, 1 µg mL^−1^ *S. mitis* B6 chromosomal DNA was added to the cultures (except non-transformed controls). Cells were harvested upon entry to stationary phase, and resuspended 1/5 in C+Y+2 µg mL^−1^ DAPI. 2 µL of these cell cultures was spotted onto a microscope slide containing a pad of 1.2% C+Y agarose as described previously [44] before imaging. Images were captured and processed using the Nis-Elements AR software (Nikon). Analysis of cell dimensions was carried out using the MATLAB-based open source software MicrobeTracker [45]. Cell contours were obtained using the *alg4 spneumoniae3* algorithm implemented in MicrobeTracker, a derivative of *alg4 ecoli2* with parameters spliltTreshold, joindist and joinangle refined to fit the shape of *S. pneumoniae*. Relative DAPI intensity was calculated by dividing the overall intensity of each cell by the area.

## Supporting Information

S1 Fig Genetic organization of the *recFOR* chromosomal regions, location of *mariner* minitransposon insertion mutants, and impact of *recFOR* inactivation on pneumococcal growth and CSP-induced competence. Chromosomal regions with insertion mutations are shown for *recF*, *recO* and *recR* respectively in panel (A), (B) and (C). Insertions (*erm*, *kan* or *spc* cassette) were located by PCR for all clones (S2 Text) and exact junctions were determined by DNA sequencing for underlined insertions. Cassettes inserted in the co-transcribed orientation are indicated by black flags. Putative transcription signals are indicated (−10) and transcription starts are shown by horizontal arrows. Primers (S1 Table) used for *mariner* insertion mutagenesis and diagnostic PCRs are indicated below each map. (D) Representative growth curves of *recFOR* mutants in C+Y medium following inoculation from frozen precultures grown until OD_550nm_ = 0.25 (mid exponential phase). Doubling time calculated within the fastest phase of growth are indicated for the wildtype strain (left) and the *recFOR* mutants (right). (E) Response to CSP of wildtype and *recFOR* mutant strains monitored using an *ssbB*::*luc* transcriptional fusion (S2 Text). Time of CSP addition is indicated by a vertical arrow. Strains used: wild type (wt), R1502; *recF* mutant, R2371; *recO* mutant, R2372; *recR* mutant, R2373; *recF*-*recO* double mutant, R2374; *recO*-*recR* double mutant, R2575; *recF*-*recO* double mutant, R2376.
(TIF)

S2 Fig *recFOR* mutants are extremely sensitive to DNA-damaging agents. (A) Sensitivity of *recFOR* mutants to methyl methanesulfonate. 20 µL spots of cultures prepared as described in S2 Text were deposited. 1 corresponds to ∼60 cfu deposited per spot. Strains used as in S1E Fig. (B) Sensitivity of *recFOR* mutants to mitomycin C. For plating conditions and strains used, see panel A.
(TIF)

S3 Fig Interplay of RecO and SsbB in plasmid transformation. (A) Impact of *ssbB* inactivation on plasmid transformation in wildtype and *recO* ^-^ cells at a high donor DNA concentration (4 µg mL^−1^ plasmid pLS1). Impact was evaluated through calculation of the ratio of transformants in *ssbB* ^-^ and in *ssbB* ^+^ cells in otherwise wildtype (R3055/R1818 ratio) or *recO* ^-^ (R3172/R3170 ratio) genetic background. Strains used: wild type, R1818; *ssbB* mutant, R3055; *recO* mutant, R3170; *ssbB*-*recO* double mutant, R3172. (B) Impact of *ssbB* inactivation on plasmid transformation in wildtype and *recO* ^-^ cells at a low donor DNA concentration (0.01 µg mL^−1^ plasmid pLS1). Same strains and calculation as in panel A.
(TIF)

S4 Fig Transformation of *glnR*::*kan* ^22C^ cassette (Kan^R^) in wildtype and *recO^-^* cells. Transformation efficiency normalized to that of the *rpsL41* point mutation (Sm^R^). Recipient strains used: wildtype, R246; *recO^-^*, R3170.
(TIF)

S5 Fig Monitoring the kinetics of disappearance of deletion junctions at chromosomal sites #2 and #3. PCRs carried out to detect deletion junctions #2 (CJ305–CJ306, panel A) and #3 (CJ307–CJ308, panel B) on cultures of wildtype and *recO^-^ xerS^-^* cells transformed with R246 genomic DNA (gDNA) and different time-points. Time point at 70 min post-DNA addition not done for junction #2 in *recO^-^ xerS^-^* cells. Strains used: wildtype; R246, *recO^-^ xerS^-^*; R3873.
(TIF)

S1 Table Strains, plasmids, and primers used in this study.
(DOCX)

S2 Table RecFOR and mismatch repair during transformation.
(DOCX)

S3 Table RecFOR and replicative plasmid transformation.
(DOCX)

S1 Text RecFOR and pneumococcal physiology, genome maintenance, mismatch repair and plasmid transformation.
(DOCX)

S2 Text Supporting Methods and Materials.
(DOCX)
