# Supplementary figures and images for: RecFOR Is Not Required for Pneumococcal Transformation but Together with XerS for Resolution of Chromosome Dimers Frequently Formed in the Process
Source: PLoS Genet. 2015 Jan 8;11(1):e1004934. doi: 10.1371/journal.pgen.1004934 (PMC4287498; doi:10.1371/journal.pgen.1004934)

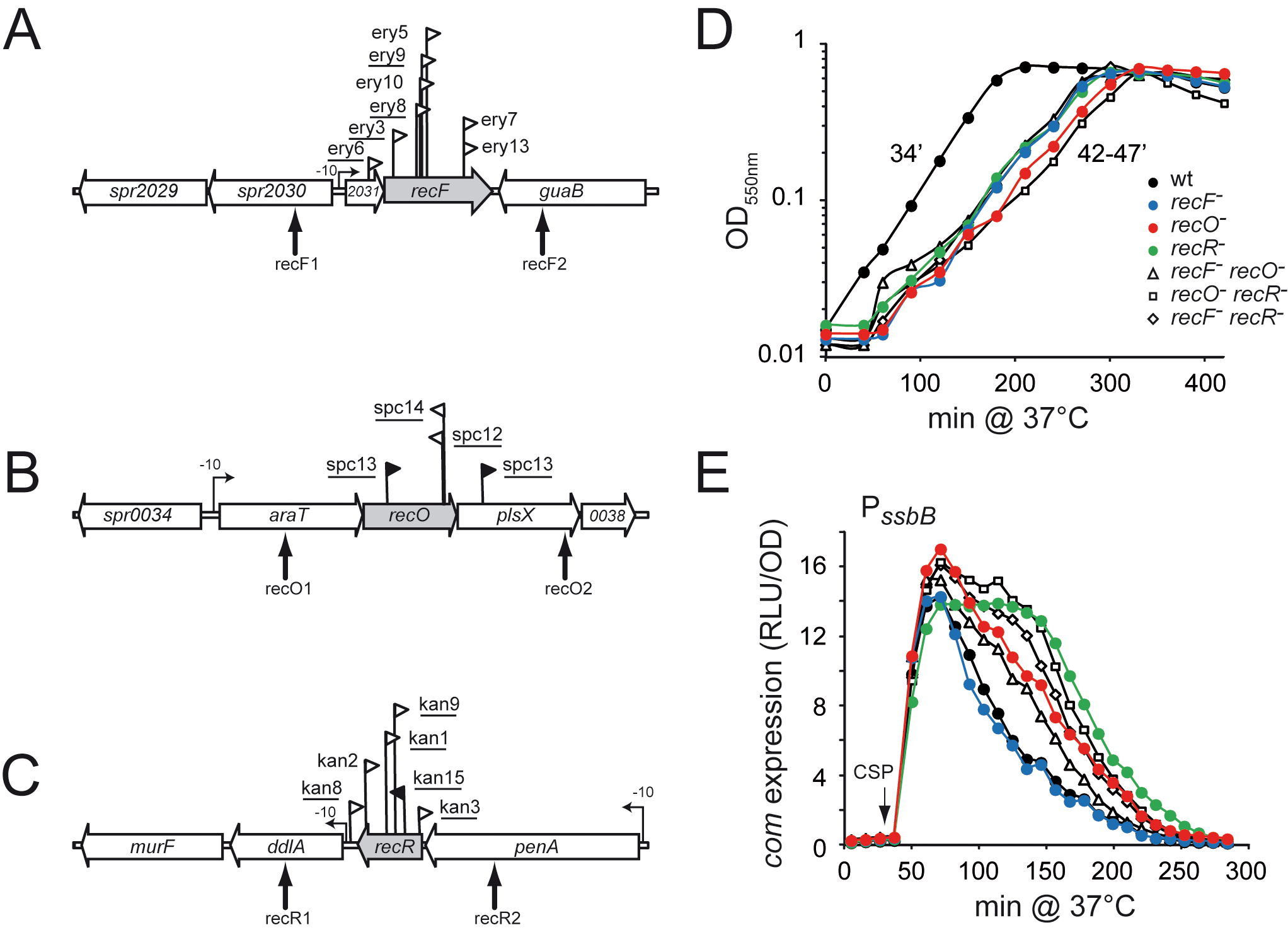

Supplement: S1 Fig — Genetic organization of the recFOR chromosomal regions, location of mariner minitransposon insertion mutants, and impact of recFOR inactivation on pneumococcal growth and CSP-induced competence. Chromosomal regions with insertion mutations are shown for recF, recO and recR respectively in panel (A), (B) and (C). Insertions (erm, kan or spc cassette) were located by PCR for all clones (S2 Text) and exact junctions were determined by DNA sequencing for underlined insertions. Cassettes inserted in the co-transcribed orientation are indicated by black flags. Putative transcription signals are indicated (−10) and transcription starts are shown by horizontal arrows. Primers (S1 Table) used for mariner insertion mutagenesis and diagnostic PCRs are indicated below each map. (D) Representative growth curves of recFOR mutants in C+Y medium following inoculation from frozen precultures grown until OD550nm = 0.25 (mid exponential phase). Doubling time calculated within the fastest phase of growth are indicated for the wildtype strain (left) and the recFOR mutants (right). (E) Response to CSP of wildtype and recFOR mutant strains monitored using an ssbB::luc transcriptional fusion (S2 Text). Time of CSP addition is indicated by a vertical arrow. Strains used: wild type (wt), R1502; recF mutant, R2371; recO mutant, R2372; recR mutant, R2373; recF-recO double mutant, R2374; recO-recR double mutant, R2575; recF-recO double mutant, R2376. (TIF) [file pgen.1004934.s001.tif]

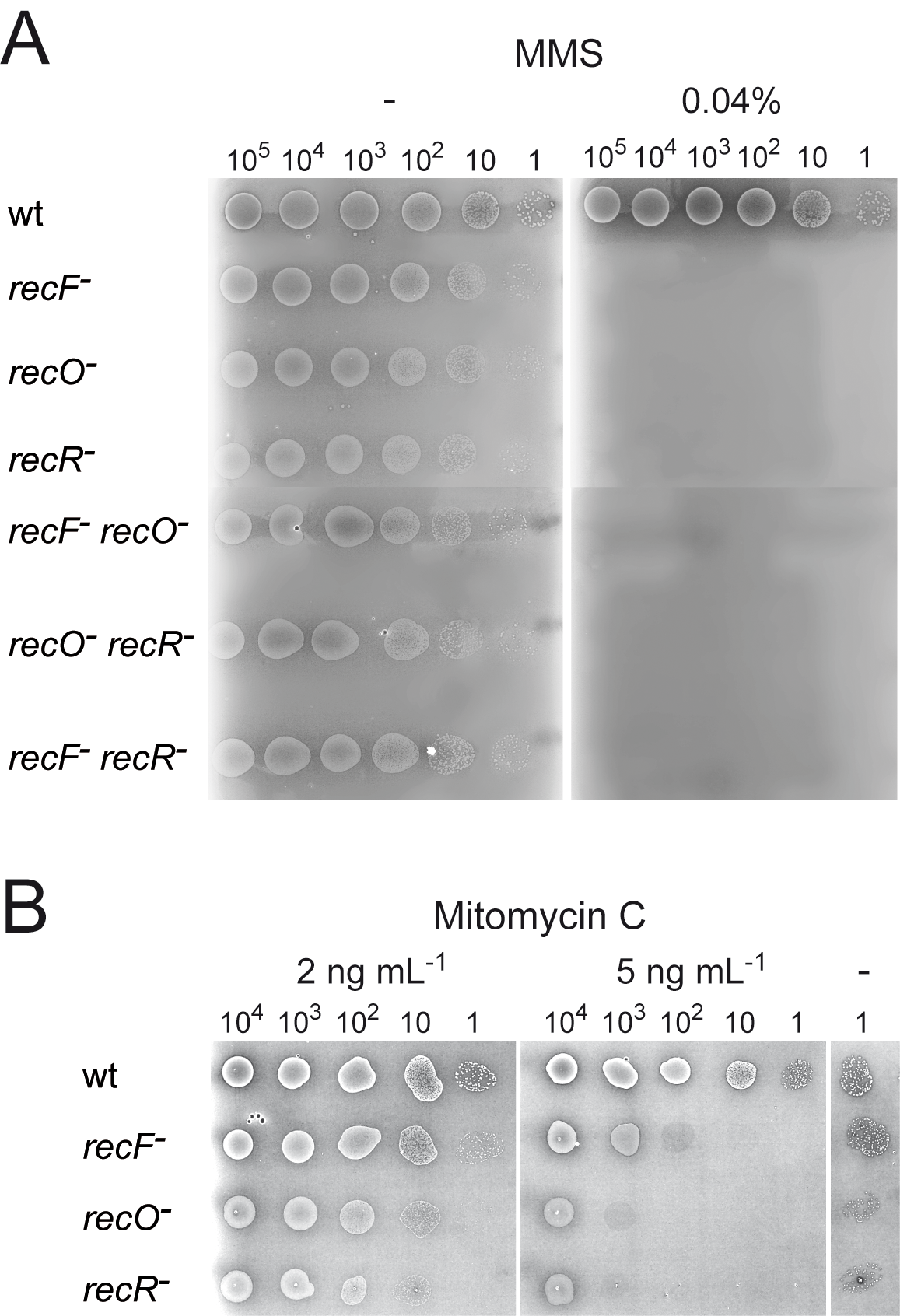

Supplement: S2 Fig — recFOR mutants are extremely sensitive to DNA-damaging agents. (A) Sensitivity of recFOR mutants to methyl methanesulfonate. 20 µL spots of cultures prepared as described in S2 Text were deposited. 1 corresponds to ∼60 cfu deposited per spot. Strains used as in S1E Fig. (B) Sensitivity of recFOR mutants to mitomycin C. For plating conditions and strains used, see panel A. (TIF) [file pgen.1004934.s002.tif]

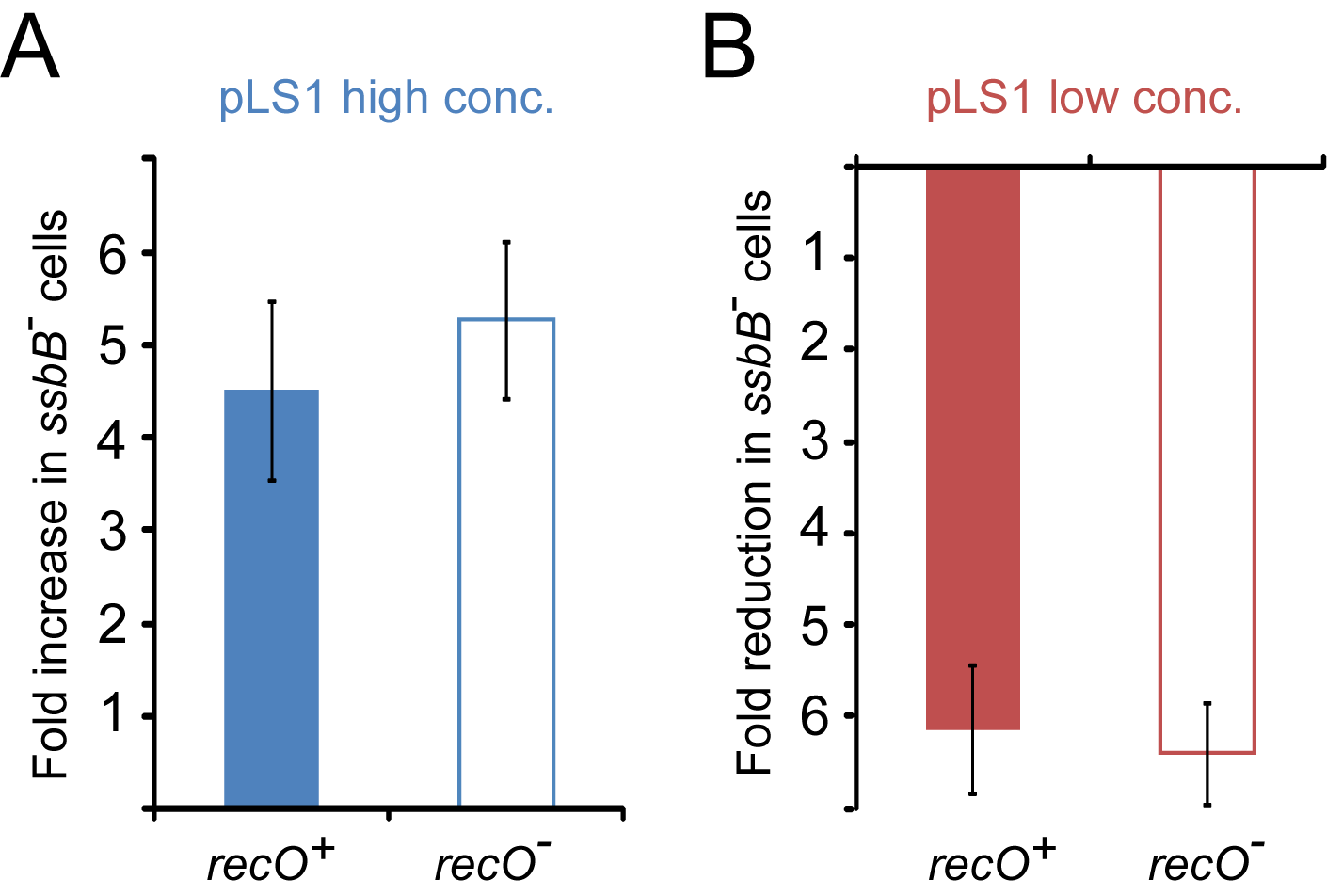

Supplement: S3 Fig — Interplay of RecO and SsbB in plasmid transformation. (A) Impact of ssbB inactivation on plasmid transformation in wildtype and recO - cells at a high donor DNA concentration (4 µg mL−1 plasmid pLS1). Impact was evaluated through calculation of the ratio of transformants in ssbB - and in ssbB + cells in otherwise wildtype (R3055/R1818 ratio) or recO - (R3172/R3170 ratio) genetic background. Strains used: wild type, R1818; ssbB mutant, R3055; recO mutant, R3170; ssbB-recO double mutant, R3172. (B) Impact of ssbB inactivation on plasmid transformation in wildtype and recO - cells at a low donor DNA concentration (0.01 µg mL−1 plasmid pLS1). Same strains and calculation as in panel A. (TIF) [file pgen.1004934.s003.tif]

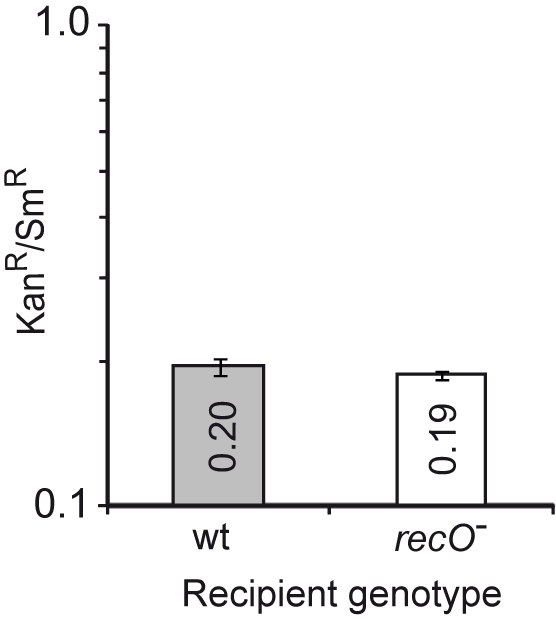

Supplement: S4 Fig — Transformation of glnR::kan 22C cassette (KanR) in wildtype and recO- cells. Transformation efficiency normalized to that of the rpsL41 point mutation (SmR). Recipient strains used: wildtype, R246; recO-, R3170. (TIF) [file pgen.1004934.s004.tif]

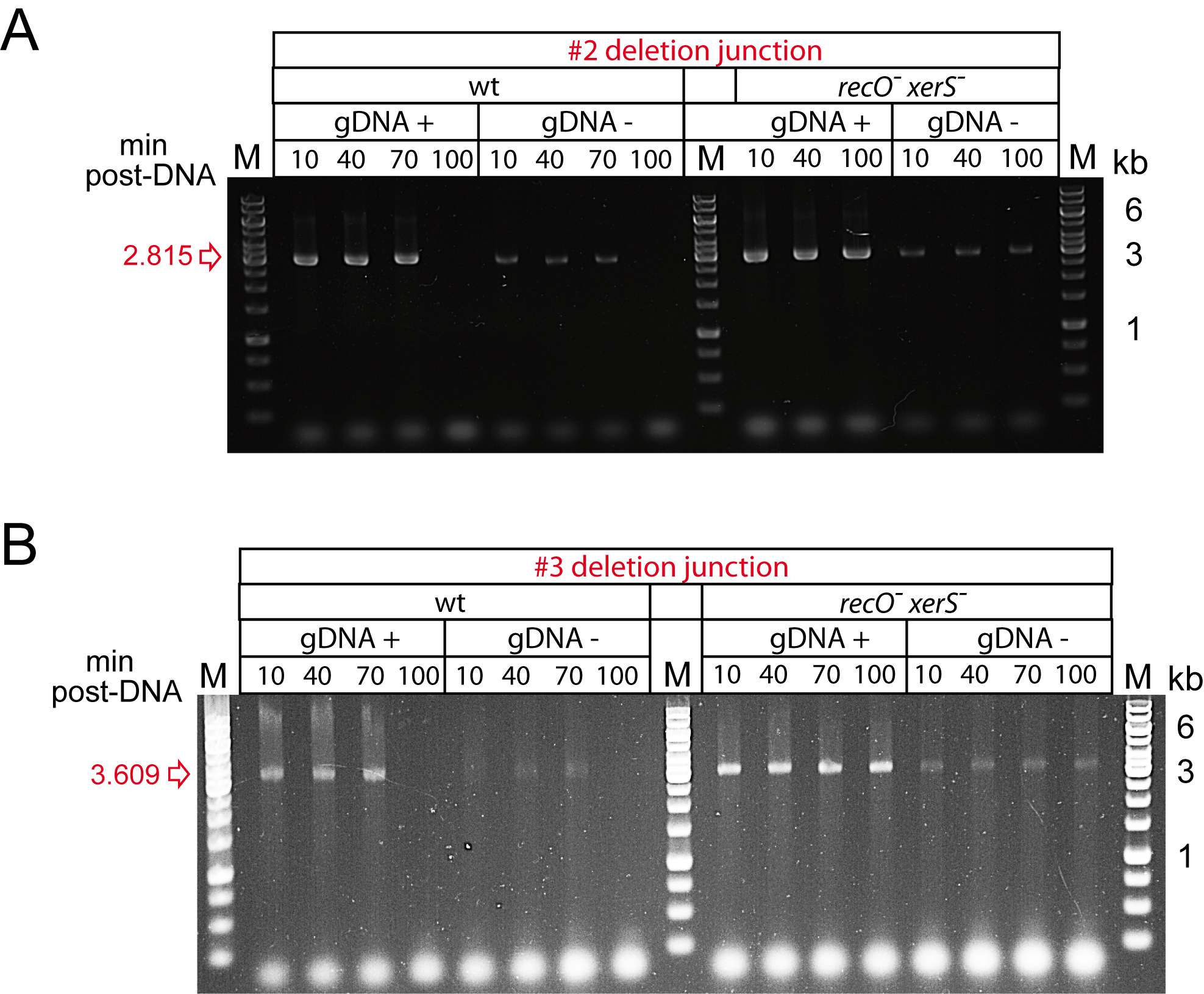

Supplement: S5 Fig — Monitoring the kinetics of disappearance of deletion junctions at chromosomal sites #2 and #3. PCRs carried out to detect deletion junctions #2 (CJ305–CJ306, panel A) and #3 (CJ307–CJ308, panel B) on cultures of wildtype and recO- xerS- cells transformed with R246 genomic DNA (gDNA) and different time-points. Time point at 70 min post-DNA addition not done for junction #2 in recO- xerS- cells. Strains used: wildtype; R246, recO- xerS-; R3873. (TIF) [file pgen.1004934.s005.tif]
