# Supplementary material for: RecFOR Is Not Required for Pneumococcal Transformation but Together with XerS for Resolution of Chromosome Dimers Frequently Formed in the Process
Source: PLoS Genet. 2015 Jan 8;11(1):e1004934. doi: 10.1371/journal.pgen.1004934 (PMC4287498; doi:10.1371/journal.pgen.1004934)
Supplement: S1 Table — Strains, plasmids, and primers used in this study. (DOCX) [file pgen.1004934.s006.docx]

**Table S1** Strains, plasmids, and primers used in this study.

| Strains | **Genotype/description** | **Source/reference** |
| --- | --- | --- |
| R246 | R800 but *hexA*Δ*3*::*ermAM*; Ery^R^ | [46] |
| R304 | R800 derivative, *nov1*, *rif23*, *str41*; Nov^R^, Rif^R^, Sm^R^ | [46] |
| R800 | R6 derivative | [47] |
| R1192 | R800 but *ssbB*::*spc^2^*^C^; Spc^R^ | [19] |
| R1501 | R800 but Δ*comC* | [48] |
| R1502 | R1501 but *ssbB*::*luc*, *ssbB^+^*; Cm^R^ | [48] |
| R1521 | R1501 but *comC*::*luc*; Ery^R^ | [49] |
| R1818 | R1501 but *hexA*Δ*3*::*ermAM*; Ery^R^ | [19] |
| R2371 | R1502 but *recF*::*erm^8^*^A^; Ery^R^, Cm^R^ | This study |
| R2372 | R1502 but *recO*::*spc^13^*^C^; Cm^R^, Spc^R^ | This study |
| R2373 | R1502 but *recR*::*kan^15^*^C^; Cm^R^, Kan^R^ | This study |
| R2374 | R1502 but *recF*::*erm^8^*^A^ *recO*::*spc^13^*^C^; Cm^R^, Ery^R^, Spc^R^ | This study |
| R2375 | R1502 but *recO*::*spc^13^*^C^ *recR*::*kan^15^*^C^; Cm^R^, Kan^R^, Spc^R^ | This study |
| R2376 | R1502 but *recF*::*erm^8^*^A^ *recR*::*kan^15^*^C^; Cm^R^, Ery^R^, Kan^R^ | This study |
| R3055 | R1818 but *ssbB*::*kan^2^*^C^; Ery^R^, Kan^R^ | [19] |
| R3154 | R1501 but *glnR*::*kan^22^*^C^, *rpsL41*, Kan^R^, Sm^R^ | [50] |
| R3170 | R1818 but *recO*::*spc^13^*^C^; Ery^R^, Spc^R^ | This study |
| R3172 | R3055 but *recO*::*spc^13^*^C^; Ery^R^, Kan^R^, Spc^R^ | This study |
| R3214 | R246 but *xerS*::*kan* [28], Ery^R^, Kan^R^ | This study |
| R3424 | R1521 but *rec0*::*spc^13^*^C^; Ery^R^, Spc^R^ | Vanessa Khemici |
| R3426 | R1521 but *dprA*::*kan^184^*^A^ [51]; Ery^R^, Kan^R^ | Vanessa Khemici |
| R3428 | R3424 but *dprA*::*kan^184^*^A^; Ery^R^, Kan^R^, Spc^R^ | Vanessa Khemici |
| R3873 | R3170 but *xerS*::*kan* [28], Ery^R^, Kan^R^, Spc^R^ | This study |
| Plasmids |  |  |
| pR201 | pBR322 derivative; Ap^R^, Tc^R^, Ery^R^ | [52] |
| pR409 | pEMcat derivative carrying a Ery^R^ (*ermAM* gene) *mariner* minitransposon; Ap^R^, Ery^R^ | [30] |
| pR410 | pEMcat derivative carrying a Kan^R^ (*kan* gene) *mariner* minitransposon; Ap^R^, Kan^R^ | [30] |
| pR412 | pEMcat derivative carrying a Spc^R^ (*aad9* gene) *mariner* minitransposon; Ap^R^, Spc^R^ | [30] |
| pLS1 | pMV158 derivative, replicative plasmid (rolling-circle type); Tet^R^ | [53] |
| pLS70 | pLS1 derivative carrying a 3480 bp *Pst*I genomic fragment from the maltose locus; Tet^R^ | [53] |
| Primers | **Sequence; gene; position^§^** |  |
| MP127 | CCGGGGACTTATCAGCCAACC; mariner transposon | [30] |
| MP128 | TACTAGCGACGCCATCTATGTG; mariner transposon | [30] |
| recF1 | TTATCAACTACTGCGGGTGAA; *recF*; -891 | This study |
| recF2 | TTATTGCTGACGGTGGGATC; *recF*; +1589 | This study |
| recO1 | ACAGGAATTACCTACAGTCGAGAG; *recO*; -632 | This study |
| recO2 | CATCGCTTGAGCCATGAGTC; *recO*; +1646 | This study |
| recR1 | ACTAGACCCCATGTTTGACGG; *recR*; +1306 | This study |
| recR2 | GGTCTTATGGGGCAAACCTA; *recR*; -674 | This study |
| rpsL5 | CTGGATCTACAAAGGCGGCT; rpsL; -2020 | [54] |
| rpsL6 | TGGCTCTTCGTCAGATGCTG; rpsL; +2229 | [54] |
| codY7 | ATTTCACCAGTCAATGCTTTCACA; *codY*; -4018 | [27] |
| codY8 | AAATGAAAACGCTTTCTAGGCAAA; *codY*; +3376 | [27] |
| CJ242 | CAGTGACGTCAAAAGCAAGGC, α ; (1,430,484-1,430,504)* | [27] |
| CJ244 | TAGCCTAGAATGTGTCGTAAACTT, γ ; (1,323,806-1,323,829) | [27] |
| CJ245 | ATGATGGACAGACCTTTACTATCCT, δ ; (1,434,193-1,434,169) | [27] |
| CJ305 | ataggtggataaaagtcttcacaa, (352,321-352,344) | This study |
| CJ306 | TAGGATAATGGTTTTTGTCTTACGTT, (499,290-499,265) | This study |
| CJ307 | accaactgtcgcttcaccaaaacgg, (974128-974104) | This study |
| CJ308 | TTCAATGGCTGATTTGTTTGGTGACA, (760903-760928) | This study |
| merod-a1 | CTACCGAAACCAGCCTCATCTTG; R_1_-A ; (1,324,548-1,324-570) | [27] |
| merod-a2 | GTATCAGGTGTTGAATGTACTGCCC; R_2_-Z ; (1,432,002-1,432,026) | [27] |
| merod-b | CCAGAACCAGAACGTGACACTGAC; R_1_-A, β ; (1,327,551-1,327,528) | [27] |
| merod-c | GAAGGACAGGCAACTTGCAGGTC; R_2_-Z ; (1,434,817-1,434,795) | [27] |

**^§^**3' oligonucleotide position given with respect to the ATG of the corresponding gene; - and + indicate upstream and downstream, respectively

*position of oligonucleotide sequence on R6 reference genome.

^C^ and ^A^ indicate respectively the co-transcribed and the reverse orientation of an inserted mini-transposon antibiotic resistance gene with respect to the targeted gene

46. Mortier-Barrière I, de Saizieu A, Claverys JP, Martin B (1998) Competence-specific induction of *recA* is required for full recombination proficiency during transformation in *Streptococcus pneumoniae*. Mol Microbiol 27: 159-170.

47. Lefèvre JC, Claverys JP, Sicard AM (1979) Donor deoxyribonucleic acid length and marker effect in pneumococcal transformation. J Bacteriol 138: 80-86.

48. Dagkessamanskaia A, Moscoso M, Hénard V, Guiral S, Overweg K et al. (2004) Interconnection of competence, stress and CiaR regulons in *Streptococcus pneumoniae*: competence triggers stationary phase autolysis of *ciaR* mutant cells. Mol Microbiol 51: 1071-1086.

49. Martin B, Granadel C, Campo N, Hénard V, Prudhomme M et al. (2010) Expression and maintenance of ComD-ComE, the two-component signal-transduction system that controls X-state (competence) of *Streptococcus pneumoniae*. Mol Microbiol 75: 1513-1528.

50. Johnston C, Martin B, Granadel C, Polard P, Claverys JP (2013) Programmed protection of foreign DNA from restriction allows pathogenicity island exchange during pneumococcal transformation. PLoS Pathogens 9: e1003178.

51. Bergé M, Moscoso M, Prudhomme M, Martin B, Claverys JP (2002) Uptake of transforming DNA in Gram-positive bacteria: a view from *Streptococcus pneumoniae*. Mol Microbiol 45: 411-421.

52. Alloing G, Trombe MC, Claverys JP (1990) The *ami* locus of the Gram-positive bacterium *Streptococcus pneumoniae* is similar to binding protein-dependent transport operons of Gram-negative bacteria. Mol Microbiol 4: 633-644.

53. Stassi D, López P, Espinosa M, Lacks SA (1981) Cloning of chromosomal genes in *Streptococcus pneumoniae*. Proc Natl Acad Sci USA 78: 7028-7032.

54. Bergé MJ, Kamgoué A, Martin B, Polard P, Campo N et al. (2013) Midcell recruitment of the DNA uptake and virulence nuclease, EndA, for pneumococcal transformation. PLoS Pathog 9: e1003596.
