# Supplementary material for: RecFOR Is Not Required for Pneumococcal Transformation but Together with XerS for Resolution of Chromosome Dimers Frequently Formed in the Process
Source: PLoS Genet. 2015 Jan 8;11(1):e1004934. doi: 10.1371/journal.pgen.1004934 (PMC4287498; doi:10.1371/journal.pgen.1004934)
Supplement: S2 Table — RecFOR and mismatch repair during transformation. (DOCX) [file pgen.1004934.s007.docx]

| **Table S2** RecFOR and mismatch repair during transformation. | | | | |  |
| --- | --- | --- | --- | --- | --- |
|  |  |  |  |  |  |
|  |  | Recipient strain | | | |
|  |  | wt | *recF^-^* | *recO^-^* | *recR^-^* |
|  |  | (R1502) | (R2371) | (R2372) | (R2373) |
|  |  |  |  |  |  |
| Rif/Str |  | 0.129 | 0.122 | 0.147 | 0.160 |
|  |  | ± 0.010 | ± 0.014 | ± 0.016 | ± 0.019 |
|  |  |  |  |  |  |
