# Supplementary material for: RecFOR Is Not Required for Pneumococcal Transformation but Together with XerS for Resolution of Chromosome Dimers Frequently Formed in the Process
Source: PLoS Genet. 2015 Jan 8;11(1):e1004934. doi: 10.1371/journal.pgen.1004934 (PMC4287498; doi:10.1371/journal.pgen.1004934)
Supplement: S3 Table — RecFOR and replicative plasmid transformation. (DOCX) [file pgen.1004934.s008.docx]

| **Table S3** RecFOR and replicative plasmid transformation. | | |  |  |  |
| --- | --- | --- | --- | --- | --- |
|  |  |  |  |  |  |
|  |  | Plasmid donor DNA | |  |  |
| Recipient strain |  | pLS1^a^ | pLS70^b^ |  |  |
|  |  |  |  |  |  |
| wt (R1502) |  | 0.015% | 0.468% |  |  |
| (R1502) |  | ± 0.006 | ± 0.06 |  |  |
|  |  |  |  |  |  |
| *recF^-^* |  | 0.004% | 0.288% |  |  |
| (R2371) |  | ± 0.003 | ± 0.044 |  |  |
|  |  |  |  |  |  |
| *recO^-^* |  | 0.028% | 0.328% |  |  |
| (R2372) |  | ± 0.010 | ± 0.056 |  |  |
|  |  |  |  |  |  |
| *recR^-^* |  | 0.023% | 0.336% |  |  |
| (R2373) |  | ± 0.0092 | ± 0.056 |  |  |
|  |  |  |  |  |  |
|  |  |  |  |  |  |
| ^a^Replicative plasmid (rolling-circle type; pMV158 derivative) (Table S3) | | | |  |  |
| ^b^Replicative plasmid (pLS1 derivative) carrying a 3486 bp-long chromosomal insert (Table S3) | | | | | |
