# Supplementary material for: RecFOR Is Not Required for Pneumococcal Transformation but Together with XerS for Resolution of Chromosome Dimers Frequently Formed in the Process
Source: PLoS Genet. 2015 Jan 8;11(1):e1004934. doi: 10.1371/journal.pgen.1004934 (PMC4287498; doi:10.1371/journal.pgen.1004934)
Supplement: S1 Text — RecFOR and pneumococcal physiology, genome maintenance, mismatch repair and plasmid transformation. (DOCX) [file pgen.1004934.s009.docx]

**Text S1** RecFOR and pneumococcal physiology, genome maintenance, mismatch repair and plasmid transformation.

**Impact of *recFOR* inactivation on pneumococcal cells**

Each *recFOR* gene was inactivated by *mariner* mutagenesis, as previously described [30] (Text S2; Figure S1A-C; Table S1). The impact of mutating these genes on pneumococcal physiology was first investigated by comparing the growth rate of single and double mutants with that of the wildtype. All mutants were found to have doubling times of between 42 and 47 min, slower than the wildtype which doubled every 34 min (Figure S1D).

The RecFOR proteins are involved in genome maintenance in various species, with mutants showing sensitivity to DNA-damaging agents (see Introduction). To determine whether this was also the case in *S. pneumoniae*, we then examined the sensitivity of the *recFOR* mutants to the alkylating agent methyl methanesulfonate (MMS) by plating serial 10-fold dilutions (from 6x10^1^ to 6x10^6^ cfu) of the wildtype, single and double mutants on the surface of blood-agar plates containing or lacking 0.04% MMS. While survival of the wildtype was not affected by the presence of MMS at this concentration, none of the mutants survived, irrespective of the number of cells plated (Figure S2A).

Next, we examined the sensitivity of single *recF*, *recO* and *recR* mutants to the DNA crosslinking agent mitomycin C, by plating serial 10-fold dilutions (from 6x10^1^ to 6x10^5^ cfu) of the wild type and these mutants on the surface of blood-agar plates containing or lacking mitomycin C (2 or 5 ng mL^-1^). Results show that wild-type survival was not affected by presence of mitomycin C, while mutant cells showed reduced viability in presence of mitomycin C in a concentration-dependent manner (Figure S2B).

**RecFOR and mismatch repair during transformation**

To determine whether loss of *recFOR* impacted mismatch repair during pneumococcal transformation, two distinct point mutations carried on chromosomal DNA were transformed into recipient cells either wildtype or lacking *recF*, *recO* or *recR*. The point mutations tested were *str41* conferring Sm^R^, and *rif23* conferring Rif^R^ [31]. It is known that the Hex mismatch repair system ejects certain point mutations during transformation, antagonizing the integration process [32]. Thus, the *rif23* point mutation is frequently ejected by the Hex system, while *str41* is not recognised as efficiently. As a result transformation of *rif23* is 10-fold less efficient than that of *str41* in wildtype cells [31].

Comparing the efficiency of transformation of these point mutations in *recF*, *recO* and *recR* mutant cells equally showed a 10-fold decrease in efficiency of transformation of *rif23* compared to *str41* (Table S2), establishing that loss of RecFOR proteins does not affect the activity of the Hex system during chromosomal transformation in *S. pneumoniae*.

**RecFOR proteins are not involved in transformation of a replicative plasmid**

Owing to the mechanism of uptake of transforming DNA, which results in the internalization of ssDNA fragments, plasmid establishment through natural transformation relies on the annealing of plasmid strands that have entered the cell separately, from two donor molecules [55],[56]. Annealing is thus crucial for reconstitution of an intact plasmid replicon. RecO is an obvious candidate for catalyzing this annealing, and plasmid transformation was reduced 25-fold in a *B. subtilis* *recO* mutant [24]. We therefore measured the transformation efficiency of a replicative plasmid, pLS1, in pneumococcal *recFOR* mutants. This efficiency was not altered by absence of RecF, RecO or RecR (Table S3), suggesting no role for these proteins in plasmid installation. The same was observed for a pLS1 derivative plasmid pLS70 (Table S3), which is replicative, but can also use homology in the recipient chromosome as a template to facilitate reconstitution of a complete molecule, hence the name facilitation of plasmid transfer [57]. Overall, these results suggest that the pneumococcal RecFOR proteins play no role in replicative plasmid transformation

However, the antagonization of plasmid transformation by the single-stranded DNA-binding protein SsbB, which is dedicated to chromosomal transformation, was previously observed in *S. pneumoniae* at a high concentration of donor plasmid DNA [19]. This effect was tentatively attributed to SsbB antagonizing RecO [19]. To check this hypothesis, plasmid transformation was carried out in parallel in wildtype, and *recO* and *ssbB* single and double mutant cells. Inactivation of *ssbB* resulted in a similar increase in transformation in both wildtype and *recO* mutant cells (Figure S3A). The increase in plasmid transformation in the absence of SsbB is therefore not due to a relief of inhibition of RecO-dependent annealing of internalized plasmid single-strands.

As RecO could be overloaded with excess internalized ssDNA at a high concentration of plasmid DNA, we further assayed plasmid transformation using a low concentration of donor DNA. Under these conditions, *ssbB* inactivation reduced plasmid transformation to the same extent in both wild type, as previously reported [19], and *recO* mutant cells (Figure S3B). Thus even with limiting substrate, there is no support for the hypothesis that SsbB could antagonize RecO-dependent annealing of plasmid strands, and therefore no indication that RecO could participate in plasmid transformation.

Taking these results altogether, we conclude that RecO is not involved in plasmid strand annealing whether SsbB is present or not, and whatever the concentration of donor plasmid DNA.

55. Saunders CW, Guild WR (1981) Monomer plasmid DNA transforms *Streptococcus pneumoniae*. Mol Gen Genet 181: 57-62.

56. Saunders CW, Guild WR (1981) Pathway of plasmid transformation in pneumococcus: open circular and linear molecules are active. J Bacteriol 146: 517-526.

57. López P, Espinosa M, Stassi D, Lacks SA (1982) Facilitation of plasmid transfer in *Streptococcus pneumoniae* by chromosomal homology. J Bacteriol 150: 692-701.
