# Supplementary material for: RecFOR Is Not Required for Pneumococcal Transformation but Together with XerS for Resolution of Chromosome Dimers Frequently Formed in the Process
Source: PLoS Genet. 2015 Jan 8;11(1):e1004934. doi: 10.1371/journal.pgen.1004934 (PMC4287498; doi:10.1371/journal.pgen.1004934)
Supplement: S2 Text — Supporting Methods and Materials. (DOCX) [file pgen.1004934.s010.docx]

**Text S2** Supporting Materials and Methods.

***In vitro mariner* mutagenesis**

Insertions of the *ermAM* (Ery^R^), *kan* (Kan^R^) or *spc* (Spc^R^) gene cassette in *recFOR* were generated by in vitro *mariner* mutagenesis as previously described [30]. Briefly, the plasmids used as a source for *mariner* minitransposons were pR409, pR410 or pR412 (Table S1). Plasmid DNA (~1 μg) was incubated with a *recFOR* fragment (~1 μg) amplified with primer pairs recF1-recF2, recO1-recO2 or recR1-recR2 (Table S1), in the presence of purified Himar1 transposase, in a total volume of 20 μL leading to random insertion of the minitransposon within the fragment. Gaps in transposition products were repaired as described [30] and the resulting in vitro-generated transposon insertion library was used to transform *S. pneumoniae*. Location and orientation of *mariner* cassettes was determined through PCR reactions using primers MP127 or MP128 (Table S1) in combination with either one of the two primers used to generate the *recFOR* PCR fragment (Figure S1A-C). Cassette-chromosome junctions were sequenced using primer MP128.

**Monitoring of growth and response to CSP of *recFOR* mutants**

To measure the response to CSP, a transcriptional fusion of the *ssbB* gene promoter to the luciferase gene (*luc*) was used to follow expression levels from this promoter as previously described [43]. For the monitoring of growth and *luc* expression, precultures were gently thawed and aliquots were inoculated (1 in 100) in luciferin-containing [43] C+Y medium and distributed (300 ml per well) into a 96-well white microplate with clear bottom. Relative luminescence unit (RLU) and OD values were recorded throughout incubation at 37°C in a LucyI luminometer (Anthos).

**Sensitivity to DNA damage**

To compare the sensitivity to mitomycin C and to MMS of a wildtype strain and recFOR mutants, stocks of bacteria grown in Todd–Hewitt (BD Diagnostic System) plus yeast extract (THY) to an OD_550_ of 0.4 were diluted 100-fold in C+Y medium (pH 6.8 to 7.0) and incubated at 37°C to an OD_550_ of 0.2. Then, 20 µL of each culture and of 10-fold serial dilutions were spotted on 1-day-old D-agar plates containing catalase (500 U mL^-1^). Plates contained variable concentrations of mitomycin C or MMS. In all cases, plates were incubated overnight at 37°C.
